# Assessment of knowledge, attitudes, and practices related to the prevalence of sheep scab among communal sheep farmers in Eastern Cape Province, South Africa

**DOI:** 10.14202/vetworld.2024.558-563

**Published:** 2024-03-07

**Authors:** Mandla Yawa, Bukeka Mtenjwa, Ishmael Festus Jaja, Siza Mthi, Nkululeko Nyangiwe, Sive Tokozwayo, Francis Rumosa-Gwaze, Thuthuzelwa Stempa, Luxolo Qokweni

**Affiliations:** 1 Department of Rural Development and Agrarian Reform, Döhne Agricultural Development Institute, Private Bag X15, Stutterheim 4930, South Africa; 2 Department of Livestock and Pasture, Faculty of Science and Agriculture, University of Fort Hare, Private Bag X1314, Alice 5700, South Africa; 3 Department of Agriculture and Animal Health, University of South Africa, Florida, South Africa; 4 Department of Rural Development and Agrarian Reform, P.O. Box 112, Queenstown 5320, South Africa; 5 Ikhala TVET College, Queen Nonesi Campus, Queenstown 5320, South Africa; 6 Department of Rural Development and Agrarian Reform, Private Bag X3090, Butterworth 4960, South Africa; 7 Department of Rural Development and Agrarian Reform, Private Bag X6012, Port Elizabeth 6001, South Africa

**Keywords:** communal sheep farmers, Eastern Cape Province, season, sheep scab control methods, sheep scab

## Abstract

**Background and Aim:**

Sheep scab is one of the most contagious diseases of sheep found in rural communities worldwide and is a major health and welfare concern for sheep farming. Information on the attitudes of communal farmers to sheep scab remains speculative in the Eastern Cape Province. This study aimed to investigate knowledge, attitudes, and practices related to the prevalence of sheep scab among communal sheep farmers in Eastern Cape Province, South Africa.

**Materials and Methods:**

From June to August 2022, a cross-sectional survey using a semi-structured questionnaire (n = 160) was conducted in two rural communities of the Enoch Mgijima Local Municipality in Eastern Cape Province. Purpose sampling was used to obtain respondents’ knowledge, attitudes, and management practices regarding sheep scabs.

**Results:**

Among respondents, 81% were males and 19% were females. The majority of sheep farmers (59%) in both communities were aware of sheep scabs, but the majority (64%) did not receive any farming training. The majority of sheep farmers (65%) believed that sheep scab was the most serious threat to their sheep production since it increased mortality. In the past 3 years, approximately 57% of respondents reported an increase in the prevalence of sheep scab. The majority of farmers reported wool loss as the most noticeable clinical symptom (73%). More than 80% of sheep farmers do not use acaricide on a regular basis. Sixty-one percent of farmers prefer a pour-on topical application method. The frequency of annual dipping was every fortnight (40%), during summer and winter (24%). Most farmers (66%) regularly vaccinate their sheep.

**Conclusion:**

The prevalence of sheep scab is increasing in these communities; however, a better understanding of the factors that facilitate its transmission is required to allow improved management and control of this disease. The government must involve extension officers and distribute them to disseminate information to farmers. Thus, it will be easy to translate the literature into action.

## Introduction

Globally, the sheep population is around 1 billion, with 19% in Asia and Africa. South Africa has approximately 8000 small commercial livestock farms and 5800 communal/subsistence farmers [1]. South Africa’s sheep population is estimated to be around 21.464 million [2]. Sheep offers a wide range of products, including meat, wool, skin, manure, and, to a lesser extent, milk, all of which contribute to the economic success of farms [3, 4]. In South Africa, sheep are mainly raised in communal areas under a largely agricultural system. The extensive agricultural system relies largely on natural resources for grazing, without effective management systems in place. In addition, there is an intensive farming system where all management systems continue to be applied and monitored. However, sheep scab is one of the main threats to sheep production in South Africa and globally. Sheep scab disease has also been reported in other countries [5].

Sheep scab (Psoroptic mange) disease is caused by *Psoroptes ovis* and is of economic importance in sheep production [6]. The first case of sheep scab disease was reported in a year of the pre-Julian Roman calendar (180 BC). The disease was brought to South Africa by early immigrants in 1809 [7]. Severe skin irritation leads to wool loss and reduced feed intake as the animals scratch and bite themselves [8]. As a result, sheep lose weight, lowering their immunity and increasing susceptibility to secondary infections, including pneumonia and parasites [9]. Farmers pay for prophylactic acaricides, and if their sheep become infected, they pay for treatment and the cost of concealing the damage and the loss in economy from abridged stock growth, lower reproductive rate, and loss of wool production [8]. The disease can easily spread across flocks and is expensive to treat; therefore, it is recommended to improve its prevention. Sheep scab usually spreads through direct contact between sheep. *P. ovis* can be spread if the infected sheep rub against fences, posts, and trees. It is recommended that good biosecurity should be used as a preventive measure to reduce the risk, particularly in the case of intensive farming. A new flock of sheep should be treated/vaccinated for scab when they arrive on a farm or are isolated for 2 weeks, and contact with infectious livestock should be avoided [10].

Sheep scabs have been identified in communities, and this disease is considered to be one of the most important threats to sheep productivity. It can cause serious economic losses and animal welfare problems [11]. When sheep lose weight, their immunity decreases, leading to the risk of secondary infection and other diseases [12]. Sheep scab has a negative economic impact since the wool of contaminated animals must be destroyed instead of being sold since scab wool is heavier than normal wool and is referred to as scab wool if it is shipped to the buyer. Infected wool is less valuable than clean wool, and the farmer or supplier will suffer financial losses [8, 13].

A sufficient understanding of sheep scab will allow farmers to diagnose the disease early and minimize further economic losses through strategic control measures. Therefore, this study aimed to investigate knowledge, attitudes, and practices related to the prevalence of sheep scab among communal sheep farmers in Eastern Cape Province, South Africa.

## Materials and Methods

### Ethical approval

This study was approved by the University of Fort Hare Research and Ethics Committee with an ethical clearance certificate number: 201923059-BM-MY.

### Study period and location

This study was conducted from June to August 2022 in MacBride (−32.070914S; 26.781135E) and Ezola (−31.917023S; 26.629628E) villages which belong to Whittlesea communities. Whittlesea is a semi-rural town situated 37 km south of Komani (previously known as Queenstown), Eastern Cape Province, South Africa. MacBride and Ezola receive an annual rainfall of 540–650 mm and a mean annual temperature of 22°C–28°C [14]. The majority of households, particularly older people, are involved in extensive agricultural farming, namely crop production and livestock production. Sheep, goats, and cattle are known to be the major livestock for investment in these communities. Natural grasslands are generally used for livestock grazing. Most of the farmers at MacBride and Ezola prefer to farm with sheep compared to other livestock animals because of the dual benefits (i.e., meat and wool production) from sheep. However, all members of the household have the same right to access the available communal resources. Therefore, the management of livestock and grazing lands has become more complex as farmers struggle to contain contiguous diseases such as sheep scab.

### Data collection

A snowball sampling technique was used for data collection, and 160 farmers (i.e., McBride, 80 and Ezola, 80) were interviewed using a semi-structured questionnaire, where at least 30% of the sheep farmer’s population participated in this study as it was once used [15]. We divided the questionnaire into three sections: Demographic information, knowledge of sheep scab prevalence, and knowledge of control methods. Face-to-face interviews were conducted by four well-trained enumerators and animal health technicians. The farmers’ preferred languages were IsiXhosa and English. The questionnaires were administered in the farmers’ preferred language. During data collection, farmers were able to freely express themselves and ask any questions.

### Statistical analysis

The recorded data were captured in an Excel spreadsheet for analysis. Farmers’ knowledge and attitudes toward the prevalence of sheep scab were analyzed using the Statistical Analysis System of 2003, version 9.1 for Windows (SAS Institute Inc., Cary, NC, USA) [16]. Frequencies were determined using the PROC FREQ procedure [16].

## Results and Discussion

### Sociodemographic summary of the respondents

Demographic and socioeconomic factors are significant because they provide a clear overview of respondents. Table-1 shows the sociodemographic characteristics of the respondents under study. The majority of households were headed by males (81%) compared to females (19%) (Table-1). This is in accordance with the findings of Mapiliyao *et al*. [17], Yawa *et al*. [18], and Mthi *et al*. [19], which reported that most communal livestock farmers are males. The status and role of men in households, especially in rural areas, maybe the reason for this dominance since women face a great deal of prejudice in African culture, in particular as regard livestock farming and inheritance. This may also be due to the ability and skills of handling and managing livestock. However, these results contradict [20–22], who reported that many females were sheep and goat farmers in Nigeria, Ogun State, Botswana, and South Africa compared with males. Small ruminants are smaller and easier to handle, and in some developing countries, women play a key role in agricultural value chain production and livestock production; they are more likely to inherit livestock due to substantial agricultural participation [23]. The results of the age of farmers indicate that the majority (43%) of farmers are still in their economic (35 and 44) active age. These results support [24] findings and suggest that sheep farming can be performed at various ages. In addition, due to the increased prevalence of communicable diseases, such as COVID-19, most people do not live up to the age of 60 [25, 26].

**Table-1:** Sociodemographics of sheep farmers in Ezola and MacBride locations.

Items	Frequency (n = 160)	Proportion (%)	Statistical significance	p-value
Location			*	0.044
EZola	104	65^A^		
MacBride	56	35^B^		
Total		100		
Gender			**	0.007
Male	130	81^A^		
Female	30	19^B^		
Total		100		
Age			*	0.039
≤18–24	26	16^C^		
25–34	45	28^B^		
35–44	68	43^A^		
45–54	21	13^C^		
Total		100		
Level of income			**	0.008
≤R 6000	104	65^A^		
R 6001–10,000	46	29^B^		
R 10,001–20,000	10	6^C^		
Total		100		
Level of education			*	0.037
No grade 12	94	59^A^		
Grade 12	36	23^B^		
Tertiary qualification	30	19^B^		
Total		100		
Farming experience			*	0.040
≤5 years	26	16^C^		
6–10 years	42	26^B^		
≥11 years	92	58^A^		
Total		100		

Significant at

* p ≤ 0.05 and

** p ≤ 0.001. ^A,B,C^Least square means in the locality with superscript differ from each other significantly (p < 0.05)

Most farmers (65%) earned less than or equal to $316.42/month, and most of the respondents relied on farming for income. These income judgments are in accordance with Mthi and Nyangiwe [27], who suggested that household income, an ever-increasing human population, and export revenues should be fulfilled to meet the stresses of household income. Sheep productivity must be increased. Education is essential to create a resilient community to the effects of sheep scab [28]. The majority of farmers (59%) have not completed Grade 12 and only 19% have completed tertiary education. Lack of education is linked to poverty and marginalization. Less-educated farmers are also more likely to experience sheep mortality due to sheep scab [29, 30]. Approximately 58% of farmers had more than 11 years of farming experience, followed by 6–10 years (26%) and <5 years (16%), 16–20 years (14%), 6–10 years (13%), and <5 years (12%). These results are in agreement with Mthi and Nyangiwe [27], Bahta *et al*. [29], and Nyam *et al*. [30], who claimed that the typical sheep farming experience is about 13 years, and while experience is gained with time, elderly people do not have to subjugate farming in any territory. The majority of sheep farmers in both areas were aware of sheep scab due to their many years of farming experience. Farmers with more than 5 years of farming experience can set realistic goals and become more committed to various farming activities [19].

### Respondents’ knowledge of the prevalence of sheep scab

**Figure-1:**
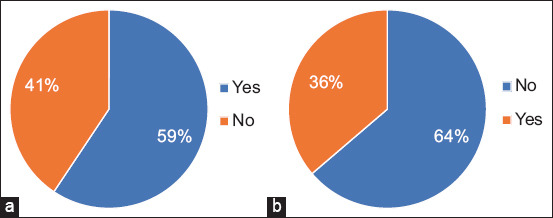
(a) Farmers knowledge of sheep scab. (b) Farmers training on sheep scab.

Figures-1a and b show farmers’ knowledge of sheep scab prevalence. The majority of sheep farmers (59%) in both communities knew about sheep scab, while 41% did not know what it was. Most sheep farmers (64%) did not receive any training in farming, which resulted in a lack of information about sheep scab control measures and poor management skills, leading to scabs becoming more common [8]. The majority of sheep farmers consider sheep scab to be the most serious threat to their sheep production since it increases mortality. These results are consistent with those reported by Mapiliyao *et al*. [17], and Anaeto *et al*. [20], who reported that the high mortality rate and prevalence of sheep scabs and parasites could be attributed to the high cost of treatment, the considerable distance traveled to healthcare facilities, and the visibility of animal health consultants. In the past 3 years, more than 57% of respondents indicated an increase in sheep scab. Sheep scab is a significant problem for sheep productivity, decreasing growth rates and leading to serious health problems. The findings of the present study are consistent with the conclusions of Watson [31] and Sturgess-Osborne [32] that the prevalence of sheep scab has continued to increase in the past 20 years since it was declassified as a notifiable infection in England and Wales in the 1990s, while it has been notifiable in Scotland since 2010.

### Farmers’ perceptions of sheep scab challenges and clinical signs

Table-2 shows the farmers’ perception of the sheep scab challenges and clinical signs. The majority of sheep farmers (65%) considered sheep scab to be the most serious threat to their sheep production because it significantly contributed to mortality. In the past 3 years, approximately 57% of respondents reported an increase in the prevalence of sheep scab. Most farmers (72.5%) noted wool loss as the most noticeable clinical symptom, followed by frequent rubbing (27.5%) on fences and walls. Approximately 85% of sheep farmers did not practice acaricide rotation. These results may be due to the fact that the majority of sheep farmers concentrate on wool production because they sell it in both communities. These results are consistent with those reported by Nixon *et al*. [6] and Murshed *et al*. [33], who reported that the predominant clinical symptoms observed in sheep infested by mange mites were itching, the animal attempting to rub the infested region with its legs or walls, fences, and some sheep biting the infested area with their teeth, as well as wool loss, and that the disease is more prevalent in winter and spring. Two acaricides, organophosphate plunge dip and endectocides, are available for sheep scab. Rotations of acaricides with diverse action approaches have been proposed as an additional efficient prevention and control strategy [32]. Acaricide rotation was not practiced by the majority of the farmers. This may be due to the cost of education and the low level of literacy in communities; the cause of illiteracy may be the financial situation of families, which leads them to make education for their children a low priority.

**Table-2:** Perception of farmers on sheep scab clinical signs and challenges.

Items	Frequency (n = 160)	Proportion (%)	Statistical significance	p-value
Clinical signs of sheep scab			*	0.035
Frequent rubbing	44	27.50^B^		
Wool loss	116	72.50^A^		
Weight loss	0	0.00^C^		
Skin sores	0	0.00^C^		
Total		100		
Mortalities			*	0.042
Yes	104	65.00^A^		
No	56	35.00^B^		
Total		100		
What are views on the prevalence of sheep scab in the past 3 years			*	0.038
Increasing	91	57.00^A^		
Decreasing	46	29.00^B^		
Not sure	23	14.00^C^		
Total		100		
Acaricide rotation			**	0.008
Yes	24	15.00^B^		
No	136	85.00^A^		
Total		100		

Significant at

* p ≤ 0.05 and

** p ≤ 0.001. ^A,B,C^Least square means in the locality with superscript differ from each other significantly (p < 0.05)

### Farmers’ perceptions on treatment systems and treatment intervals

Table-3 shows the farmers’ perceptions of the treatment systems and treatment intervals. Sixty-one percent of farmers preferred a pour-on topical application technique, whereas a small percentage of farmers used a plunge dip tank (23%) and spray (16%). The frequency of annual dipping was every fortnight (40%) during the summer and winter seasons (24%). The majority of farmers (66%) vaccinated their sheep, and the most frequent vaccination intervals were seasonal (53%), annual (38%), and monthly (10%). Due to an increase in the prevalence of sheep scab, approximately 61% of communal farmers elected to utilize pour-on dipping at intervals of every fortnight, monthly, seasonal, and weekly; however, limited farmers use spray and plunge dip tanks, which may be due to resource shortages such as water. The majority of farmers vaccinate their sheep and the most frequent vaccination intervals are seasonal, yearly, and monthly. The high price of vaccines is the primary reason for the increased mortality rate of sheep, and the lack of funds among smallholder farmers may explain why 44% of farmers do not vaccinate sheep [27].

**Table-3:** Perception of farmers on treatment systems and treatment interval.

Items	Frequency (n = 160)	Proportion (%)	Statistical significance	p-value
Dipping systems			*	0.043
Plunge	36	22.50^B^		
Pour-on	99	61.25^A^		
Spray race	0	0.00^C^		
Hand dressing	26	16.25^B^		
Total		100		
Dipping interval			*	0.032
Weekly	8	5.00^C^		
Fortnightly	64	40.00^A^		
Monthly	50	31.25^AB^		
Seasonal	38	23.75^B^		
Total		100		
Sheep vaccination			*	0.048
Yes	105	66.00^A^		
No	55	34.00^B^		
Total		100		
Vaccination interval			*	0.036
Monthly	16	10.00^C^		
Seasonal	84	53.00^A^		
Yearly	60	38.00^B^		
Total		100		

Significant at

* p ≤ 0.05. ^A,B,C^Least square means in the locality with superscript differ from each other significantly (p < 0.05)

## Conclusion

Sheep farmers consider sheep scab to be the most serious threat to their sheep production because it increases mortality. Due to the increasing prevalence of sheep scab, farmers in both areas were aware of sheep scab. Several sheep farmers did not receive any type of farming training, which resulted in a lack of knowledge about sheep scab control strategies and poor management skills, which increased the prevalence of scab. Farmers used pour-on most throughout the dipping process. This was attributed to resource scarcity (e.g. water), which limited the use of pour-on rather than alternative dipping methods such as plunge, which is known to be the most effective external parasite control method. Sheep scab seriously threatens sheep productivity, reduces growth rates, and causes serious health problems. The government should involve extension officers and distribute information to communal farmers. Thus, it will be easy to translate the literature into action. It is necessary to provide contributions to all farmers, such as the supply of acaricides, so that all community farmers are able to control this disease without fear of financial damage.

## Authors’ Contributions

MY, BM, and IFJ: Identified the research topic and study area and drafted the manuscript. SM, FR, NN, TS, LQ, and ST: Analyzed and interpreted the data and revised the final manuscript. All authors have read, reviewed, and agreed to the published version of the manuscript.
